# Relationship between type 2 diabetes mellitus and cognitive decline: A community‐based 4‐year prospective cohort study

**DOI:** 10.1002/alz70860_099098

**Published:** 2025-12-23

**Authors:** Liangjun Dang, Yi Zhao, Ling Gao, Shan Wei, Chen Chen, Kang Huo, Suhang Shang, Jin Wang, Qiumin Qu

**Affiliations:** ^1^ The First Affiliated Hospital of Xi'an Jiaotong University, Xi'an, Shaanxi, China; ^2^ The First Affiliated Hospital of Xi’an Jiaotong University, Xi’an, shaanxi, China; ^3^ The First Affiliated Hospital of Xi’an Jiaotong University, Xi’an, China; ^4^ The First Affiliated Hospital of Xi’an Jiaotong University, Xi’an, Shaanxi, China

## Abstract

**Background:**

To examine the impact of type 2 diabetes mellitus (T2DM) on cognitive decline.

**Method:**

This was a community‐based prospective cohort study. The population was selected using clustering sampling method and people who lived in the Qubao village of Xi’an aged 40 years or older were all enrolled. The people with normal cognition were surveyed between October 2014 and March 2015 as baseline, and two follow‐up visits were conducted in 2016 and 2018. Standardized questionnaires were employed to collect data on individual characteristics, lifestyle, medical history, and physical and biochemical assessments. Participants were categorized based on T2DM medical history and fasting blood glucose levels into three groups: the pre‐existing T2DM group, the new‐onset T2DM group during follow‐up, and the non‐T2DM group. Cognitive function was assessed using the Mini‐Mental State Examination (MMSE). Cognitive decline was defined as a drop of ≥2 points in MMSE score over the 4‐year follow‐up. Chi‐square tests and multivariate logistic regression were used to evaluate the effect of T2DM status on the 4‐year risk of cognitive decline.

**Result:**

A total of 1,350 participants were included in the analysis. 1,096 (81.2%) were free of T2DM, 158 (11.7%) had pre‐existing T2DM, and 96 (7.1%) developed new‐onset T2DM during the follow‐up period. Cognitive decline was observed in 230 participants (17.0%). The incidence of cognitive decline had significant difference among the three groups (non‐T2DM vs. pre‐existing T2DM vs. new‐onset T2DM: 15.7% vs. 20.9% vs. 26.0%, *p* = 0.014). Multivariate logistic regression revealed that the risk of cognitive decline was not significantly different between the pre‐existing T2DM group and the non‐T2DM group (OR=1.402, 95% CI: 0.890‐2.210, *p* = 0.145). However, the new‐onset T2DM group exhibited a significantly increased risk of cognitive decline than non‐T2DM group (OR=1.726, 95% CI: 1.029‐2.896, *p* = 0.039).

**Conclusion:**

New‐onset T2DM had significantly higher risk of cognitive decline over 4 years among individuals aged 40 years and older in rural Xi’an. These suggest that the early stage of T2DM may be a critical period for the cognitive decline.

